# Extra Supplement—The Nursing Mirror

**Published:** 1890-11-01

**Authors:** 


Being the Extra Nursing Supplement of "The Hospital" Newspaper.

All Contributions for this Supplement should be addressed to the Editor, The Hospital, 140, Strand, London, W.C., and should have the word "Nursing" plainly written in left-hand top corner of the envelope.

### En Passant.

TO OUR READERS.—An enlarged Mirror is offered to our readers to-day, which we hope will reflect more fully and more faithfully all the events and opinions which sway the nursing world. Our readers have increased in number; our foreign correspondence is growing steadily; and it is evident that more space is only the due of those many friends of ours who take the trouble to write to us. The Mirror is practically run by nurses for nurses: unless our readers sent us the news we could never obtain it so correctly and swiftly, and our best thanks are due to those who, in the interest of their profession and their fellow workers, avail themselves of our pages to circulate far and wide all news that concerns and is helpful to nurses. On our side we do our best to answer the many letters and queries sent us, to lend a helping hand where one is needed, and to supply that information which not only amuses but instructs. Many a busy matron puts aside her ledger to yield us information, many a smiling sister welcomes us to see the improvements in her wards, and nurses and probationers grant us a portion of their hardly-earned off-duty time. And thanks to these, we hope and believe that the image we reflect is not altogether unworthy or incorrect. It is no ideal nurse, free from all fault, who is painted in these pages. "Know thyself," said the old philosopher, and the nurse, above all women, needs this counsel—her aims are so high, her failures so disastrous. There are not many of us who can "be true to every inmost thought," or who can act day by day up to the high standard held before us. It is no true kindness to describe every nurse as an angel; it is far better to help her to see the best way to become one.

THE FALL.—We have never echoed in these pages the *furore* of praise showered at one time on Sister Rose Gertrude. Knowing somewhat about her early career during the period she was at Winchester, and while she was acting as secretary to Mr. Harry Quilter, we thought the greatest kindness to the poor girl was to keep silence, and to hope that the Catholic religion would help her to carry out a life of sacrifice for which she had otherwise seemed unsuited. Public credulity saw nothing incongruous in the fact that a young girl should go to Molokai with every intention of superseding, not only the Sisters of Mercy who have laboured silently there for years, but also the medical staff; but every nurse knew that the scientific study of the leprosy germ was not compatible with the ordinary duties of a woman sworn to be a servant of the sick. The *Nightingale* says: "So arrogant have been her pretensions that technical difficulties are developed which hinder her entry upon her chosen field. The little Sister tarries about, and in the meantime cruel reports appear in the papers to the effect that the gifts entrusted to her for the lepers have never been delivered." There is a still more cruel report about, that Sister Rose Gertrude is going to marry Dr. Lutz, an avowed agnostic. What is truth and what not we cannot say, but this is evident—that the fall has come. The flight was too high for the poor Sister, and the notoriety has been dearly bought. The sad part is that the shame falls on the nursing profession as a whole; how shall we get the public to believe in and support the next mission to the lepers, while the failure of Sister Rose Gertrude is before their eyes? The higher the flight the more disastrous the fall; let every nurse, then, hide deep in her heart her great ambitions, and tread upward slowly and surely, striving not beyond her strength in public places, unless she would bring ignominy on herself and her calling.

TYNEMOUTH INFIRMARY.—This is a charming little hospital, with great possibilities for good work and nurse-training, but at present it seems to miss part of its vocation. The committee is composed chiefly of working men, and, strange to say, they seem to consider the comfort of the patients less than do those who have never had experience of illness in hospital wards. The utmost economy is observed in all departments. A short time ago, at the advice of Dr. and Mrs. Mears, a nurse-matron was obtained and probationers procured, but the probationers have resigned, and all attempt at nurse-training given up. Private nurses are badly needed at Tynemouth, and for this, as well as for the poorer class of patients, the infirmary might be made a centre of usefulness. The people of Tynemouth should spare no effort to increase the efficiency of the infirmary and remodel those departments which are not so successful as they might be.

NURSES AT NORWICH.—At the quarterly meeting of the Norfolk and Norwich Hospital, on October 17th, the Rev. Canon Feilden called the attention of the Board to the scheme for using The Shrubbery, which adjoins the hospital, as a residence for the nurses connected with the scheme for providing trained nurses to sick persons outside the hospital. He said the committee which had been appointed to consider the question had not been able to complete its deliberations, although they had held sittings. The present tenant of The Shrubbery, Mr. J. H. Tillett, treated the committee in a most generous and handsome manner, and had written offering to make his arrangements coincide with the best interests of the hospital. By his kindness he had removed what might have been a great difficulty. There is no doubt that Norwich will soon be able to choose in the matter of private nurses between the old-established and very favourite staff of nurses at 50, Bethel Street, and the nurses who will shortly be supplied by the hospital.

SO IRISH, YOU KNOW!—The Limavady Board of Guardians have appointed a "young lady" as nurse at the infirmary, at a salary of £15. This "young lady" had been a nurserymaid, and did not possess any previous knowledge of or training in nursing, and the Local Government Board refuses to think she is a proper person to have sole charge of the unfortunate sick paupers. The whole story is quite archaic, and forcibly impresses upon us how far behind in these matters is the beautiful Emerald Isle. Perhaps the saddest thing about the matter is the tone of a leader in a local paper; it is this paper which persistently describes the nurse (?) as a "young lady" possessed of all the virtues, and, in fact, far too good to serve "a lot of pauper patients, many of them reduced to that condition by their own bad conduct and dissipated habits." This sort of tone is most reprehensible. We have no doubt that the whilom nurserymaid is an intelligent and respectable girl, and is doing her best to secure the well-being of those under her charge. We are quite willing to grant all this to her champion, but we would bid him remember that nursing is an art, and like everything else must be learnt. This editor would not leave an errand boy with no previous experience of journalism in charge of his paper; this editor would not go to a hospital porter for a prescription for sciatica. A little common sense would show the Guardians that they had better accept the suggestion of the Local Board quietly, and pay £25 a year to a trained nurse. Doubtless, they did not formerly consider the matter sufficiently; there are no lady guardians at Limavady, and gentlemen cannot be expected to be so well up in these matters. A little thought must show them that it is a matter of life and death to place an inexperienced young girl in charge of the sick and dying; it is very hard that the dying should not have the most skilled attention during their last few sad hours or days on this earth, and those who have been "sick unto death" know well what agony the tending of untrained hands can cause, and what comfort can be gained from the ministering of the nurse skilled in her work.

### Lectures on Surgical Ward Work and Nursing.

By Alexander Miles, M.B. (Edin.), C.M., F.R.C.S.E.

Introduction.

In the course of lectures on surgical ward work which I begin this week, it is not my intention to treat of the subject of surgery proper, but rather to confine my attention to some of those practical clinical points, which are, I think, too often neglected in the teaching alike of nurses and medical students.

These papers are intended mainly for junior nurses beginning their surgical work, and for those who have not had an opportunity of attending a systematic course of lectures on this subject during their hospital training. That they may also prove instructive to medical students entering on their duties as clerks and dressers in the wards of our surgical hospitals I am not without hope.

I propose not only to state the duties of a surgical nurse, but, as best I can, to enter into the details of these duties, explaining as far as is necessary for their intelligent performance the why and the wherefore of each.

I am afraid there are many things to which I shall have occasion to refer, which may appear to you very trivial, and almost too self-evident to necessitate their being dwelt upon, but as it is just these points which are usually omitted by teachers, and which are at the same time the most valuable for you to know, I must ask you to bear with me while I speak of them.

If I do enlarge too much on the details and minutiæ of ward work, it is because I am convinced that it is only by the most careful attention to these, that a surgical nurse can hope to attain proficiency in her profession, and that only in so far as she is conversant with the minute particulars of her work is she a competent nurse or the reverse. "There is no real elevation of mind in a contempt of little things."

It matters little to a poor man who has met with a serious accident, whether or not his nurse be acquainted with the various indications for, and methods of amputating at the hip-joint, but it is of importance to him that she can properly adjust his dressings, change his sheets without causing him discomfort, or serve up his food so that he takes it with a relish; and let me remind you that it is the latter duties that are yours.

### Lecture I.—ANTISEPTICS.

There are three words which every surgical nurse hears constantly used in the wards, and of which it is of the very first importance that she should thoroughly understand the meaning.

These words are "Septic," "Aseptic," and "Antiseptic." There are many technical and scientific terms used by the doctors which it is of little importance that you, as nurses, should understand, but it is not so with the three constantly recurring words which I have just mentioned. And if it is necessary that you should understand the words themselves, it is of infinitely greater import that you should fully enquire into, and intelligently grasp, the principles of that system of treating surgical wounds in relation to which they are used.

I shall therefore first try to bring before you the general principles on which "Antiseptic Surgery" is based.

The word "Septic" is derived from the Greek verb "septo" I putrify, hence when we speak of a septic wound we mean a wound in which the process of putrefaction is going on. We must then enquire, What is this process, and under what conditions may it arise? Now, the series of chemical changes which we term sepsis or putrefaction, requires for its production two main factors; the one existing in the patient himself, to wit, *a wound*; the other coming from without—*micro-organisms.*

We shall first then consider *Micro-organisms.* The first great fact I would ask you as surgical nurses to get firmly fixed in your minds, and never again to forget, even for a moment, is that you and your patients are everywhere and always surrounded by myriads of micro-organisms, and that it is these that are the causes of sepsis.

The term "Schizomycetes" has been applied to the whole range of micro-organisms, and includes all the various forms of bacteria, bacilli, micrococci, vibrios, etc. In less scientific language they are spoken of as germs or microbes, but perhaps the most convenient term for us is that of "micro-organisms."

*Size, Shape, Colour.*—These micro-organisms vary greatly in their size, shape, and colour. They are all so small as to require high powers of the microscope to render them visible; and very powerful lenses are necessary for the investigation of their structure and life changes. In shape also they present very many varieties—round, oval, rod-shaped, comma shaped, spiral, and so on. In several species the protoplasm of which they are composed exhibits traces of pigmentation, the prevailing colours being yellow, orange, red, and blue.

*Habitat.*—As I have already said, these organisms are practically omnipresent. To obtain an idea of their abundance you have only to recall the amount of dust which is rendered visible in the air by a ray of sunshine entering a darkened room, and to remember that each dust particle is literally covered with them. And if they are thus so abundant in the atmosphere it is evident that they will be more or less prevalent under almost every conceivable condition of ordinary life, in the water we drink, on our clothes, our furniture, and even on our skin.

The number of organisms in the atmosphere depends on the height from the earth, the temperature, the amount of dust, and the proximity of material, such as decomposing animal matter, on which they live.

*Structure.*—Each organism is really a small cell, consisting of a minute mass of protoplasm bounded by an envelope, and in some cases coloured by one or other of the pigments before mentioned.

*Life changes.*—While they differ in many points, such as shape and colour, they all agree in their mode of life, and in possessing the power of setting up putrefaction, when placed under favourable conditions. For their nutrition they require a supply of hydrogen, nitrogen, oxygen, and carbon, and most require some phosphate, and to obtain these they break up the elements of the tissues in which they find a "nidus," or suitable habitat; this breaking-up process being what we know as putrefaction or sepsis.

The most indispensable condition to the septic process is, that the tissue on which the germ settles be devitalised—"below par." Few or none of these organisms can attack with success a tissue which is healthy, hence it has been said, and with truth, "the best antiseptic we possess is healthy tissue." Here then is a valuable indication in the treatment of surgical patients. If you want to prevent sepsis or to counteract it, begin at the very foundation and get your patients tissues into as healthy a condition as possible, by attending to his diet, ventilation, exercise, excretory functions and so on, and so by rendering the nidus unsuitable starve out the enemy. It is because a surgical wound is of necessity in a somewhat devitalised condition, that it is so easily attacked by germs.

Of the other conditions necessary to the life of these organisms, moisture and heat are the most important, and both of these are found in an ordinary wound. They grow best at a temperature of about 96·8 deg. F., hence the temperature of the body, 98·4 deg. F., they find fairly comfortable.

Having thus glanced at the nature and causes of putrifaction we are in a position to use the word "septic" intelligently. This being so it follows that the words aseptic and antiseptic become easily understood; the former (from *a* not, and *septo*, I putrify), meaning that the wound is free from putrefaction; the latter (from *anti*, against, and *septo*), indicating that the substances to which it is applied are actually capable of counteracting sepsis or putrefaction.

*(To be continued.)*

### Six Months in a Hospital.

By a "Special Pro."

V.—*(Conclusion).*

I was ordered off this morning to prepare the isolated ward for a special operation case—ovariotomy—and all the day I have been airing sheets, filling pillows, scrubbing tables, and heaven alone knows what else.

If you have absolutely nothing to do for the next three months, and any money you are anxious to get rid of, you can make some little flannel night gowns for the children here. They are very simple of course, and vary from infant's white night gowns up to any size almost. Perhaps when you come and see some of the patients and go over the wards, you will feel some slight enthusiasm kindle in your breast.

I am in the accident ward to-day, with a suicide case. The man got a razor on Thursday midday while his wife and family were away, also a pail to receive his blood. He cut his throat fearfully in two places, after which he shut up the razor, and put it away on a shelf, covered with blood. He managed to divide the trachea by cutting so deeply. So a tracheotomy tube has been inserted in the wound, and the case is really now a tracheotomy case, with the unusual accompaniment that the patient performed part of the operation himself! It is a sad case. Poor, out of work, depressed in mind, and sick in body, and tired out of the whole thing called in courtesy "life." It seems almost cruelty to nurse him back again to his misery! I am "special" to him from nine a.m. to nine p.m., and another nurse does corresponding duty at night. We have two cut-throats here, one a convalescent. On Thursday we had two "bobbies" in the ward, one for each case. My "bobby" was very nice; showed me the razor; and also the proper way of handling a policeman's truncheon, for as he had just brought the case in of course he was in uniform. Now we have only one, who comes on duty for eight hours and is then relieved by another. They are such nice, handy men, will do anything for you, from washing up mugs to squeezing out hot sponges. We have got such a romantic-looking, handsome fellow in our ward, and so nice; it is quite a pleasure to do anything for him. I have come to the conclusion that "blue blood" is a delusion and a snare; one meets with it in labourers, and horse keepers, and "all sorts and conditions of men" if you get the right sort.

My cut-throat is getting on well, though he has a hole as large as a small reel of cotton in his throat still. Eats rabbit, pudding, &c., quite well now. We had a lovely case in —— ward yesterday, though a frightfully sad one.

A poor fellow, working in a tunnel, was crushed between some timber. His head was bent right down between his knees by a bar across his shoulders, and in consequence his spine was fractured. He felt no pain at the time of the accident, and none below the seat of the injury at any time; nor ever will for ever more I fear, being paralysed fatally through the blow on the spinal cord. Has lost all sensation below the waist. Isn't it dreadful? We got him on to a huge water-bed yesterday. Three porters lifted him in a sheet, but I fear nothing will save his poor back from bed-sores. Another man in the accident ward (my ward to-day) had his arm caught in a saw-mill, three fingers torn off, and elbow dislocated. Strange to say, he also felt no pain, nor even knew he was injured until he went to lift his arm up, and found he had lost the power of doing so! I am still doing relief duty, some days working like a horse, and then getting grumbled at because I haven't done enough; other days less to do. I am a regular "pro," you know, now, so these letters must stop.

### Words of Consolation.

### THE GREAT PHYSICIAN.

You are lying, dear friend, in a sad plight, weary with pain, sick and suffering. Your aching brow, your throbbing wounds will not for an instant stop their agony. How gladly we would give you ease, but we cannot; all earthly means have been tried and Nature must take its own way. We are full of sympathy for your trouble; how can we assure you of this? To tell you thousands more people are as bad or worse than yourself would bring little relief. It would not take away one pain or help to bear your burden. The pity may for a moment give a sense of warmth and comfort to your heart, but soon you will fall back to the old disquiet. The doctors can do no more; you are getting hopeless. Courage, my friend, there is hope for all. Have you forgotten the Great Physician who can heal all your infirmities, both of body and soul? How often sin and sickness both come together. It is the old, old story. Man the sinner, God the saviour. Bear with me while I try to draw you to the footstool of grace, and talk a little while of the true Healer, the Wonderful Comforter, Who Himself bears our griefs and carries our sorrows. He is full of love for the children of men. Dwell on this love which is always around us. He keeps us by night as well as by day, and is always doing us good. Our life's path is strewn with God's mercies and gifts, "like sand upon the great sea shore." Can these afflictions be mercies, you say; are they the tokens of His love; these hard marks of the iron which is entering into our souls do they show His affection for us? Yes; they are one and all true tokens of His love. He wants you to feel your need of Him, to know in your heart of hearts that from Him you hold your very life.

When you were in health you were careless perhaps and did not seek Him, now He seeks you in chastisement that He may draw you closer to His bosom. He has laid His hand upon you, do not try to wrest yourself from His grasp. Lie still in His arms, He loves you; believe that He doeth all things well. Only believe! All things are possible to him that believeth. It seems very cruel of the surgeon to dress and probe your wound, or cut off a diseased limb, but by and bye you realise that he was cruel only to be kind. You must have died but for his skill; now, though robbed of a limb, you will be able to mix with your fellow creatures with ease and comfort.

So in a fever, you hate the nauseous medicine, the severe measures which are necessary for your cure, but as health returns you find, that out of the bitter has come the sweet, and are grateful.

God is acting in the same way through the body towards your soul. He wants it to be strong and healthy, and fit for His kingdom where nothing sinful and impure can enter. Christ tells us to cut off our right hand or our right foot if we are offended thereby, and when we do not do it for ourselves He takes away our power of sin till He brings us into His Heavenly Kingdom.

In the meantime He will not always be correcting. He will help us bear with cheerfulness the crosses He lays upon us. These afflictions which are only for the moment will seem light under His loving smile. We know the smile is there though it is too dark to see it, or we are too blind to distinguish it. He will whisper in our ears, "It is I, be not afraid." My grace is sufficient for you, for My strength is made perfect in weakness.

### Nurses on Their Travels.

### PRIVATE NURSING IN PARIS.

THE openings for private nurses in Paris are not very plentiful, and for obvious reasons they are difficult to secure. There are three institutions—the Levick Home, the St. George's Private Nursing Association, and Madame Neilsen's Institution. The first is managed on much the same lines as similar institutions at home; the nurses are paid a fair salary and have no risk, for they live in the Home when not at a case. It is extremely doubtful whether this can be of much advantage to a nurse; the remuneration does not exceed £30 a year, and that can be got in England, where it goes further and is more easily saved. The professional advantages may count for something, but not much, for nowadays practice at home and abroad is much the same.

The St. George's Nursing Association is composed of a few nurses, who club together for their rooms, take the cases in rotation, and provide for all other expenses separately.

There are other groups of nurses similar to this who have not attained to the dignity of a name, but their method is the same. The combination can have their rooms more cheaply, the rotation of cases insures equal distribution, and each individual keeps her own earnings. There are many nurses in England who would like to try and better themselves by going to the continent; for their benefit, and for the purpose of enabling them to weigh the pros and cons of such a step, the following details will be of use.

In the first place it cannot be too strongly represented to those who, from want of health or any other reason, are not thoroughly trained the folly of seeking continental employment, under the delusion that competition there is less keen than at home. The experiment has been tried frequently, with the invariable result—failure. Besides a good training, including, if possible, a knowledge of monthly nursing, anyone intending to practise on their own account must have a connection and a good knowledge of the French language and French ways.

The income of a private nurse is ten francs a-day—about eight shillings—or 300 francs (£12) a-month, provided she is occupied every day of the month. On the other side of the balance-sheet the figures are correspondingly large. For a single room the usual rate is 50 francs a-month; if a sitting-room is desired for the accommodation of visitors and possible clients, the rent will be 75 or 80 francs. First breakfast—a cup of café-au-lait with bread and butter taken in the early morning—lighting, fuel, and laundry expenses run up the landlady's bill to about 100 or 110 francs per mensem. Then as to food, when there is no case in hand, a good *déjeuner* and *diner* will cost about five francs daily if taken at a restaurant, possibly less if the nurse is familiar with French marketing and cooks her own meals. Say that the 150 francs can be made to include dress, stationery, postage, 'bus and train fares, etc., then the total expenditure for necessaries will be about 260 francs. It is obvious that for the first year or so one cannot expect to have more than six months' work, giving an income of 1,800 francs. While at her cases the rent of the rooms must be paid, and that, at the rate of 80 francs a-month, makes 480 francs for the half-year. During the six months' idleness a sum of 260 francs for necessaries, as shown above, mounts up to 1,560 francs, so that the total expenditure for the year is 2,040 francs. The balance is on the wrong side by nearly ten pounds.

The only means of curtailing expenses from the above estimate is in the matter of rooms, if one is willing to live in a single room—and she must have that as a fixed address for letters and inquiries even when at a case—Then the rental is reduced by 360 francs per annum, and the expenditure comes down to 1,680 francs, leaving a balance in hand of 120 francs.

It will be observed that no luxuries are included in this estimate, nor is any allowance made for sickness or unpunctual payments.

Most of the nurses now practising in Paris have at some time been on the staff of the Hertford British Hospital, and while there had opportunity for learning the language and customs of the French, as well as for cultivating a connection, which become useful when they launch out on their own account. The indication seems plain, but the difficulty is to follow it. The staff of the British Hospital is small, changes do not frequently occur, and the number of candidates is very great.

It is evident, then, that the position of a private nurse is not easy of attainment; it is precarious and risky when attained, unless she is well enough known to become a general favourite with the doctors on account of her thorough competence. When this desirable condition of prosperity is reached, she is able to save a good deal of money; for not only does her income increase, but her expenditure diminishes as she gets more work to do. This prospect is further brightened by the residence in such a beautiful city as Paris, or moving south with one's patients during convalescence, but its glitter should not blind the eye to the initial difficulties.

The "International Institution" is the name of that presided over by Madame Nielsen. It is situated in the Rue des Acacias, and was founded in 1885 after the failure of Mrs. Holland's Home. The management at first and for two years was on the co-operative system, but, owing to various causes—the most prominent was unprofessional jealousy and its results—that system was abandoned. Madame Nielsen engages about fifteen nurses, at the rate of £28 per annum, and the nurses, who must be certificated, have to engage for two years. The sum of half a franc is given for each day a nurse is on duty.

Madame Nielsen and her sister, together with a Swedish nurse, undertake massage and midwifery practice, and there are a number of rooms set apart for those, as well as for medical and surgical cases in the institution.

As regards personal advantages to a nurse, this is almost on a par with the above-mentioned (Levick) house.

### Death in Our Ranks.

ALICE HAMPSHIRE, for eight years Ward Sister of the Jenny Lind Ward, Royal Southern Hospital, Liverpool, died October 18, 1890. Sister Hampshire's death will be long and keenly felt by very many. She was a devoted nurse, and her kind, genial nature can never be forgotten.

### Ward Tattle.

THE head nurse will despise and discountenance gossip and tattle, and will teach all her nurses to abstain from it, never allowing them to tattle about their patient, or to listen to those who do. She will remember, and teach her nurses to remember, that the necessary confidences of the sick, as to their diseases, their personal history, their family life and troubles, are part of their misfortune, and are to be respected. If they come to her knowledge, she will hold them sacred. She will encourage her nurses to avoid disputes and jealousies among themselves; to settle their little differences frankly before they grow and get the upper hand; to help each other; to pull together, not apart.—*American Handbook.*

### Everybody's Opinion.

*[Correspondence on all subjects is invited, but we cannot in any way be responsible for the opinions expressed by our correspondents. No communications can be entertained if the name and address of the correspondent is not given, or unless one side of the paper only be written on.]*

### THE BRASSEY HOLIDAY HOME.

SISTER FROST writes: We have much pleasure in sending you a short account of our home. The home has now been open six months, and during that time we have received 171 visitors. We have had two picnics during the season, and they were quite a success. The last picnic we had in September, spending the day at Ecclesbourne and Fairlight Glen (about five miles from the home). Landaus were provided for those who wished to drive, the others walking by the cliff. Arriving at our destination in time for luncheon, we unpacked the hampers on the top of the hill, where we had an extensive view of the country. Here we did full justice to the good things our hampers contained. After this, our company dispersed, to meet again in the Glen for tea at four o'clock. Our nice white tablecloth and tempting repast was spread on the grass, which provoked envious remarks from passers-by, who wished that they could join us. We hope next year to be able to tell you of more than two picnics during the season. We are glad to add that we have had a very successful season, and have now had time to see that our home is thoroughly appreciated by those for whom it was opened, and many have gone away hoping to revisit us at some future time. We have also to thank many kind friends for their help in sending us gifts of game, fruit, and flowers, also cheque for £25 to help towards furnishing the home, which has been most gratefully accepted. We also beg to tender our thanks to you for allowing us space in your widely-circulated paper to make our home known, which has helped us very much.

### MALE NURSES.

"PECKHAM" writes:—I was exceedingly pleased to see the article written in HOSPITAL for October 4th by a male nurse concerning the rules attached to "The Hamilton Association," and can thoroughly endorse all he says, having myself been a nurse on behalf of the above Association for two years, when I paid 2s. 6d. entrance fee; but upon being desired to pay 40s. as "caution money" in accordance with the new rules, I refused to submit to this demand upon my slender purse, and was consequently dismissed; notwithstanding, I had received excellent testimonials from cases nursed by me which the Association had read. The question I should like to have answered, is, "Why was a nurse of two years' standing, and of blameless character, suddenly called upon to hand over 40s. as further proof of his honesty, and because he refused, was instantly struck off the roll?

### A DISCLAIMER.

NURSE NANCIE writes—In the spring of last year I entered a well-known London hospital as special probationer for a period of six months, and after serving my specified term, with the addition of four months, I left hospital life for a time and returned home. A few days ago, however, my attention was drawn by a constant reader of your paper to a series of letters appearing weekly in the columns of THE HOSPITAL, and which were describing with considerable detail my experiences at the institution referred to; and as the accuracy of some of the information led naturally to the supposition that I was their author, I am receiving judgment accordingly. Had the writer confined herself to generalities I should have taken no notice of the articles, but, unfortunately, the "special" representing myself is made to utter one or two personal remarks concerning both the matron and her fellow-nurses. As these articles have now become public property, I should like to be allowed to state in the same paper in which they appeared, that they have been published not only without my consent but without my knowledge.

### Appointment.

QUEEN VICTORIA INSTITUTE.—The Edinburgh branch of the Jubilee Institute has appointed Miss Mackay as district nurse at Campbeltown, and Miss Caris as district nurse at Port Glasgow; both trained by the Institute. Nurse Mackay has the distinct advantage of knowing Gaelic.

### Presentations.

MR. L. P. GIBSON, principal house surgeon to the Royal Portsmouth Hospital, has accepted an appointment in the service of the Khedive, to open the first native hospital in Upper Egypt. The nurses of the staff of the Portsmouth Hospital have just marked their esteem and goodwill by making Mr. Gibson a handsome present.

NURSE VERE, one of the oldest nurses at the Newcastle Infirmary, who, after training there and being night superintendent for one year, and subsequently sister-in-charge of medical wards for a further period of two years, sailed last week for Canada, where she has accepted a post in Miss Gee's Nursing Home, Montreal. Before her departure she was presented with a handsome travelling bag from her fellow-nurses, as well as a beautiful silk shawl from the patients in her ward, together with other numerous and useful presents, accompanied with the good wishes of all connected with the hospital.

### Vacancies.

THE following vacancies are announced:

**Nurses.**

Bedfordshire Institute, Bedford.

Burton-on-Trent Institution; £25.

Barony Parochial Hospital, Glasgow (night); £24.

Bradford Fever Hospital; £26.

Belper Union; £25.

Bournemouth Institution.

Blackheath and Richmond Institutions.

Chester Diocesan Deaconess Institution; £24.

Chester Union; £25.

Chest Hospital, Victoria Park; £20.

Dorset County Hospital (night); £22.

Darlington Fever Hospital; £27.

Eastern Fever Hospital, Homerton; £30; Assistant, £22.

Eston Hospital, near Middlesboro'; £25.

Easter Ross Combination Poorhouse; £30.

General Nursing Institute, Covent Garden.

Grimsby Hospital.

Hampshire Institute, Southampton.

Hanover Institute, W.; £25.

Hull Home.

Isolation Hospital, Tolworth (Head); £75.

Jessop Hospital, Sheffield (night); £25.

Leeds Institution.

Lloyd Cottage Hospital.

Jersey Institute; £25.

Middlesboro' Fever Hospital.

Manchester Ship Canal Hospital (night); £25.

Military College, Oxford.

Nottingham and Notts Institution.

Nursing Institute, Newport, I.W.; £20.

Oswestry and Ellesmere Cottage Hospital; £25.

Royal Albert Edward Infirmary, Wigan; £25.

Rural Nursing Association.

St. Mary's Home, Brighton; 18s. weekly.

Salop Infirmary; £25.

Stephen Monckton Home.

Sheffield Union (assist.); £20.

St. Saviour's Infirmary, East Dulwich; £20.

Seamen's Hospital, Greenwich; £20.

St. Asaph Union; £25.

St. Leonard, Shoreditch, Infirmary; £20; assist., £17.

Victoria Home, Bournemouth; £25.

Worthing Institute; £25.

West Bromwich Hospital (night); £25.

Windsor Royal Infirmary; £25.

Wilson Institution, Wimpole Street, W.; £30.

**Probationers.**

Cottage Hospital, East Grinstead.

Consumption Hospital, Bowdon; £12.

Jessop Hospital, Sheffield.

Lowestoft Hospital.

Nurses' Home, Bengeo, Herts.

St. Leonard, Shoreditch, Infirmary; £10.

Sheffield Union; £10.

**District Nurse.**

Gainsboro' Nursing Association.

**Midwife.**

Western Dispensary, Westminster.

**Superintendents.**

Convalescing Fever Hospital, Gore Farm, Darenth; £40.

Hospital for Children, Great Ormond Street; 100 gs.

### Notes and Queries.

**Answers.**

*E. C. D. L.*—Classes are being arranged. Write to the Secretary of the St. John Ambulance Association, St. John's Gate, Clerkenwell, for full particulars

*H. T.*—See "Help in Sickness."

*Homes for Incurables.*—The case mentioned by A. N. would probably be taken at All Hallows Hospital, Ditchingham, near Bungay, Suffolk.

*British Medical Association.*—In reply to E. L. H., the annual meeting of the British Medical Association was held this year in Birmingham, on July 29th, 30th, and 31st, and August 1st. Full reports of the proceedings will be found in the *British Medical Journal* of August 2nd and succeeding numbers.

*Nursing in Buenos Ayres.*—"Muriel" should write to Miss Eade, matron of the British Hospital, Buenos Ayres, enclosing a formal application for employment as a nurse, and stating where she was trained, her experience, age, and the amount of wages asked for.

*Nettle Rash.*—"Nurse P " in answer to query No. 4, writes: Nettle rash may be caused by mechanical irritation, cold, acidity, and other disorders of the stomach. The rash often appears instantaneously in the cold and disappears in the warmth. Or it may be chronic.

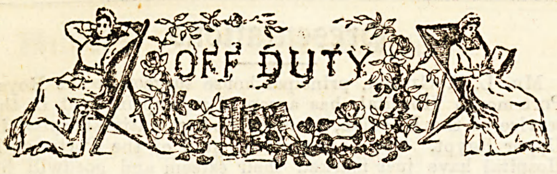

### The Hare-lipped Baby.

It was the time of year when day and night got married; I mean when Spring is giving way to Summer. Spring is a young, delicate woman; Summer is a strong, passionate man. The birds were singing a hymeneal hymn, for the birds sing all night long at that season.

The hospital nurse heard the birds singing while she padded splints in the ward of the country hospital where she was being trained as a probationer. Her head nodded over the work, for she was young, and unaccustomed to keep awake all night. Beside her stood an iron cradle, and in the cradle was a hare-lipped baby.

"Take care the child does not cry," the doctor had said, yawning while he spoke. "If it cries, the pin may come out of its lip; and then we can do no more for it."

So the girl had placed her chair close beside the iron cradle. She padded splints as she listened to the children.

Twenty beds were arranged beside the walls of the room; in each bed was a child, asleep. Sometimes a child cried, oftener a child threw off the blanket; but the ward was very still, and the nurse could hear the birds singing outside the hospital in the garden.

At last she fell asleep. The splint she was padding dropped off her knee and rolled on to the floor. Her hands fell on her white apron. She dreamt of the home she had left, her mother, and her brothers; she saw the house where she had been born, and the village churchyard where her father lay buried.

She was only asleep five minutes—no more. When she woke up she heard a child crying. It was the hare-lipped baby.

She started up, and lifted the child out of the iron cradle. But it was too late. The pin had fallen out of the child's lip, and the lip gaped.

She bent down to examine the child's face, and the baby looked at her. His eyes said, "What have you done to me?"

He was an old-looking careworn baby, the child of miserable, half-starved parents. He did not mean to look reproachful. But the girl thought that he said to her:

"I was born heavily handicapped. and you, you have added to the burden. I shall be pointed at when I go to school, I shall be scoffed at when I am a man, the woman I want to marry will refuse to have me, because you neglected your duty. Sorrow and shame will come to me, and when I die my hare-lip will be the badge by which I shall be remembered. You will not see me, you will forget my existence; but I shall live as you have made me, and I shall suffer. I may give way to despair, or jealousy leading to murder. Then men will punish me; but it will be you they ought to blame, because you who made me, what I shall be, a man with a hare-lip, repulsive and hideous."

The baby's eyes closed, but they had sent their message straight to the nurse's heart—a message that would live there for ever.

She told me the story herself; and she said, "It happened long years ago, but I remember it still. I am a hospital matron, a 'success' in the little world of nurses; but I sometimes dream of it, and I think that one day a man will come to me, and will ask 'Why did you give me this hare-lip?'"

Many a Spring has been married to many a Summer since the nurse fell asleep beside the iron cradle, many a hymeneal hymn has been sung by the birds over the seasons; but that reproachful look on the baby's face—planted there by its miserable, half-starved parents—lives on in the matron's heart. The hare-lipped baby may be dead; but if he is a man and suffering, he ought to know the truth, which is that no cruel word, no careless act, no mean thought, goes unpunished. Even the reproachful look of a hare-lipped baby leaves its sting; for remorse is like an adder, and the sting of reproach is sharper than the fangs of a serpent.

JOHN LAW.

### Lectures and Amusements.

SOMERVILLE CLUB.—On November 11th Dr. Wyld will lecture on Hypnotism, and Dr. Kate Mitchell will take the chair. On November 25th Mrs. Symes Thompson will give an address on Hygiene for Women.

CYPRUS SOCIETY.—A course of Ambulance lectures for Ladies are being given on Saturday afternoons at 10, Lower Sloane Street, under the auspices of this Society. The course began last Saturday.

ZOOLOGICAL SOCIETY OF LONDON.—Meetings for scientific business will be held on November 4th and 18th, at 8.30 p.m. at 3, Hanover Square, W.

WORKING MEN'S COLLEGE.—The free lectures for November, delivered on Saturdays at 8.30 p.m., will be by Miss Jane Harrison (to-night), Mr. Sedley Taylor, Rev. W. Page Roberts, Mr. D. F. Schloss, and the Rev. A. Boyd Carpenter.

LOLESWORTH CLUB, COMMERCIAL STREET.—On November 11th Dr. Stephen Mackenzie will open a debate on our hospital system.

THE PARKS.—The show of chrysanthemums in some of the parks just now is very fine. We advise our readers to visit Finsbury Park, Southwark Park, or the Inner Temple Gardens one day this week between 10 and 5.

MISS ANNESLEY KENEALY, Lecturer to the National Health Society, began on Wednesday, October 22nd, a course of lectures on Sick Nursing, at Hatfield, which have been arranged by Lady Florence Cecil. Lady Salisbury and many other ladies well-known in society will attend these lectures. Miss Kenealy, by request, will also give a similar course at Windsor after Christmas, which will be attended by the Princess Christian, whose interest in nursing work is well-known.

THE second lecture of the session at the Midwives' Institute and Trained Nurses' Club, 12, Buckingham Street, Strand, will be given on Friday evening, November 7th, at a quarter to eight, by Dr. W. S. A. Griffith. Subject: "The conditions of pregnancy, parturition, and the puerperium necessitating the help of a doctor." Part II. Non-members desiring to be present are requested to make early application for tickets of admission (6d. each).

### Amusements and Relaxation.

### N.B.—THIRD QUARTERLY WORD COMPETITION
### Commenced Oct. 4, 1890; ends Dec. 27, 1890.

Three prizes of 15s., 10s., 5s., will be given for the largest number of words derived from the words set for dissection.

N.B.—Word dissections must be sent in WEEKLY not later than the first post on Thursday to the *Prize Editor*, 140, Strand, W.C., arranged alphabetically, with correct total affixed.

The word for dissection for this, the FIFTH week of the quarter, being "HEROINE."

| Names. | Oct. 23rd. | Totals. | Names. | Oct. 23rd. | Totals. |
|---|---|---|---|---|---|
| Jenny Wren | 24 | 125 | Liz | 15 | 83 |
| Tinie | — | 55 | Checkmate | — | 76 |
| Agamemnon | 24 | 122 | Silver King | 22 | 98 |
| Patience | 22 | 120 | S. Anthony | — | 76 |
| Ecila | 22 | 119 | Quackah | 14 | 75 |
| Lightowlers | 20 | 115 | Reynard | 23 | 85 |
| Rouge | — | 89 | Sally | — | 27 |
| Wyamaris | 24 | 105 | Success | — | 61 |
| Qu'appelle | 24 | 105 | Caledonia | — | 52 |
| Nosam | 24 | 107 | Nurse Emma | 17 | 66 |
| Nurse Hilda | — | 44 | Hazel | — | 20 |
| Lady Betty | 21 | 104 | Pallas | — | 48 |
| Grenelle | — | 43 | Puss | — | 15 |
| Daisy | 20 | 96 | Shakespear | 18 | 67 |
| H. A. S. | 24 | 93 | Melita | 21 | 105 |
| A. B. C. | — | 66 | | | |

